# Cone beam computed tomography guided online adaptive radiotherapy for cervical Cancer: Two-year single-Centre implementation and clinical experience

**DOI:** 10.1016/j.tipsro.2026.100442

**Published:** 2026-09-27

**Authors:** Danique L.J. Barten, Karin Goudschaal, Karel A. Hinnen, Rianne de Jong, Jan Wiersma, Niek van Wieringen, Zdenko van Kesteren, Arjan Bel, Angelique van Vlaenderen, Sandy Loopeker, Bradley R. Pieters, Jorrit Visser, Colette B.M. van den Broek

**Affiliations:** a Amsterdam UMC, location University of Amsterdam, Department of Radiation Oncology, Meibergdreef 9, Amsterdam, the Netherlands; b Amsterdam UMC location, Vrije Universiteit Amsterdam, Department of Radiation Oncology, De Boelelaan 1117, Amsterdam, the Netherlands; c Leiden University Medical Center, Department of Radiation Oncology, Postbus, 9600 Leiden, the Netherlands; d Cancer Center Amsterdam, Imaging and Biomarkers, Amsterdam, the Netherlands

**Keywords:** Cervical cancer, Online adaptive radiotherapy, CBCT, Workflow evaluation, Implementation

## Abstract

**Purpose:**

Managing inter- and intrafraction motion remains a major challenge in cervical cancer radiotherapy. This study reports a two-year, single-centre experience with daily CBCT-guided cervical online adaptive radiotherapy (oART), evaluating the workflow feasibility of a radiation therapist (RTT)-led adaptive workflow, including treatment session duration and target coverage.

**Materials and methods:**

Thirty patients with cervical cancer were treated with CBCT-guided oART between October 2023 and December 2025. After initial supervised sessions, treatment delivery transitioned to an RTT-led workflow supported by a traffic-light protocol with on-call multidisciplinary backup. Cervical oART was introduced in phases, progressing from single-isocentre cases to more complex treatments including two isocentres and para-aortic lymph nodes. Workflow evaluation was based on retrospective analysis of session logs, process timing and oART workflow-related interventions.

**Results:**

A total of 744 adaptive sessions were analysed. Median total session duration was 33.0 min (interquartile range 13.0). Imaging and beam delivery times were stable, whereas decision-intensive steps showed greater variability, requiring corrections to AI-generated contours in all patients. Median total session duration was comparable between the first five supervised sessions and the subsequent RTT-led sessions (32.5 vs. 32.0 min). Contour review showed the strongest correlation with total session duration (ρ = 0.73). Uterus displacement outside the PTV due to intrafraction motion was observed in approximately 11% of all post-treatment CBCT-verifications and was typically limited to small fundal regions.

**Conclusion:**

Daily CBCT-guided cervical oART can be efficiently implemented within an RTT-led workflow, demonstrating workflow feasibility while maintaining target coverage despite intra-fraction anatomical variability.

## Highlights

• Clinical implementation of CBCT-guided oART for cervical cancer.
• Competency-based RTT-led workflow evaluated.
• Median treatment session time of 33 min.
• Target coverage maintained during daily adaptation.
• Workflow analysis identified opportunities for optimization.

## Introduction

External beam radiotherapy combined with brachytherapy is the standard of care for patients with locally advanced cervical cancer [1], [2]. A major challenge in cervical radiotherapy is interfraction anatomical variability, mainly due to changes in bladder filling, rectal distension, and bowel movement. This variability leads to shifts and deformations of the uterus and cervix between treatment sessions, which can compromise target coverage if not properly managed [3], [4]. Intrafraction motion is generally smaller but is clinically relevant [5], [6], [7], [8].

To mitigate interfraction variability, many centres have implemented the library-of-plans (LoP) approach [7], [9], [10], [11], [12], [13]. In this strategy, several pre-treatment optimized plans are generated for anticipated bladder filling variations. The most appropriate plan is selected for each treatment session using daily cone-beam CT (CBCT) imaging. Although LoP improves robustness, it has limitations: treatment planning is labour-intensive, the available plans may not adequately match the anatomy of the day, and replanning during the treatment course is frequently required [9], [11], [13], [14], [15]. For example, Reijtenbagh et al. reported replanning with a new CT scan in 18% of patients treated with a LoP protocol [11]. In addition, workflow events, such as bladder emptying or rectal venting can further reduce workflow efficiency [5].

Online adaptive radiotherapy (oART) enables daily re-contouring and plan re-optimization. Both magnetic resonance (MR) guided and CBCT-guided oART platforms rely on automation and artificial intelligence (AI) for contouring and deformable image registration. Daily oART has been implemented for several pelvic malignancies [16], [17], including cervical cancer using either CBCT-based [18], [19], [20], [21] or MRI-guided imaging [22]. By adapting treatment plans to the anatomy of the day, oART for cervical cancer has the potential to reduce the planning target volume (PTV) margins, while maintaining clinical target volume (CTV) coverage, and sparing organs at risk (OARs) [20], [21].

Despite promising early reports [18], [19], data on the workflow feasibility of CBCT-guided oART for cervical cancer in routine clinical practice remains limited, particularly regarding efficiency, target coverage and staff involvement.

This study reports a two-year, single-centre experience with daily CBCT-guided oART for cervical cancer. The aim of this study was to evaluate the efficiency and workflow feasibility of a radiation therapist (RTT)-led oART workflow. Workflow feasibility was assessed by registering treatment session duration, workflow events requiring intervention, and the ability to complete daily online adaptation within routine clinical practice while maintaining target coverage.

## Materials and methods

### Patient cohort

Between October 2023 and December 2025, 30 patients with cervical cancer were treated with CBCT-guided oART on the Ethos™ system (Varian Medical Systems, a Siemens Healthineers company, Palo Alto, Ca, USA) and included after written informed consent. The study was approved by the institutional Medical Ethics Committee (reference 2025.0921). Patient characteristics are summarized in supplementary Table A1.

Treatments were planned in line with the EMBRACE II protocol [23]. The standard treatment regimen consisted of 45.0 Gy in 25 fractions. Ten patients received a simultaneous integrated boost (SIB) to pathologic lymph nodes to a total dose of 55.0 or 57.5 Gy.

Eligible patients had primary cervical carcinoma scheduled for curative pelvic external-beam radiotherapy, with subsequent brachytherapy. Patients with a prior hysterectomy or bilateral hip prostheses were excluded.

### The oART introduction for cervical cancer

#### Introduction phases

CBCT-guided oART was introduced at our institution in 2020 as part of a staged disease-site implementation program. Before implementation for cervical cancer, substantial experience had been gained with oART for rectal [16], bladder [17], [24],breast cancer [25], [26] and palliative bone metastases [27], [28].

Cervical oART was introduced in three phases. Phase 1 included patients eligible for treatment on the Ethos system with a single isocentre (maximum cranio-caudal PTV length 22 cm), without pathological lymph nodes. Phase 2 expanded the workflow to include simultaneous integrated boost (SIB) to pathological lymph nodes. Phase 3 included all cervical cancer patients, including those requiring para-aortic or inguinal lymph node radiotherapy, with treatment plans with two isocentres (maximum cranio-caudal PTV length 36 cm).

#### RTT training program

The traffic-light protocol and training programme previously developed for rectal and bladder oART [16], [17], were adapted for cervical cancer (Supplementary fig. S1). RTTs received dedicated training in target delineation from a radiation oncologist (RO) and practised the complete oART workflow using anonymized cases on the Ethos online emulator (versions 1.1 and 2.0; Varian, a Siemens Healthineers company, USA). After training, RTTs completed ten supervised clinical sessions before transitioning to independent practice.

#### Presence of staff during sessions

An RO and medical physicist expert (MPE) supported oART sessions, and from patient 14 onwards also a technical physician (TP), to reduce workload for the RO and MPE. For the first treated patient, the RO and MPE were present at all sessions. After multidisciplinary agreement on workflow safety and RTT competency, established through structured evaluation meetings and five supervised sessions per patient, the workflow transitioned to RTT-led delivery. The RO, MPE, and TP remained available for consultation according to the traffic-light protocol or upon RTT request.

#### Offline review and feedback

During implementation, the RO reviewed the target contours of sessions 6–10 offline, representing the first five RTT-led sessions. Thereafter, weekly offline review by experienced oART RTTs provided feedback, with contour deviations discussed during regular training meetings.

### Treatment preparation and treatment planning

All patients underwent standardized treatment preparation, including implantation of three vaginal fornix fiducial markers for cervix localization during treatment [9], oral contrast administration, and planning CT and MRI acquisitions were performed with a comfortably filled bladder following a drinking protocol of approximately 0.3 L of water. Intravenous contrast was used when renal function permitted.

Target volumes (GTV and CTV) were delineated according to EMBRACE II guidelines [23]. The initial gross tumour volume (GTV-T_init_) was defined as the macroscopic primary tumour on MRI. The initial high-risk CTV (CTV-T_HR_) comprised the GTV-T_init_ and the remaining cervix, while the initial low-risk CTV (CTV-T_LR_) included the parametria, entire uterine corpus, and 20 mm of vagina inferior to the HR boundary, edited to anatomical limits. Elective nodal regions (CTV-E) were delineated conform to EMBRACE II. Intrafraction motion of CTV-T_LR_ was reported to be mostly in the cranial and dorsal direction [5], [8]. To account for intrafraction motion and to allow for small delineation errors during the oART workflow, for all patients the PTV margin for CTV-T_LR_ was 10 mm cranially and 7 mm in the other directions. CTV-E was expanded isotropically by 5 mm according to EMBRACE II. Pathological lymph nodes were contoured as GTV-N(#) and expanded isotropically by 5 mm to generate PTV-N(#) for SIB.

OARs included the bladder, rectum, bowel, sigmoid colon, bilateral femoral heads, spinal canal, ovaries (when transposed), and normal tissue. OAR contours were generated manually or using AI-assisted contouring (Annotate, Therapanacea, Paris, France), depending on software availability, and were reviewed and edited as required. From patient 14 onwards, AI contouring was performed using the integrated Ethos functionality.

A clinically approved reference plan was generated according to institutional planning objectives and EMBRACE II guidelines. The reference plan served as the basis for daily online adaptation. Treatment planning was performed using sliding window IMRT with 6 MV flattening-filter-free beams. Planning templates were adapted to target extent and treatment complexity, including single- and two-isocentre plans. For treatment plans with two isocenters, automatic feathering was used to mitigate the effect of motion between the delivery of the beams of the two isocenters. VMAT was not used because online optimization times were considered too long [29]. Reference plans underwent independent dose calculation (Mobius3D v3.1, Varian Medical Systems) as part of quality assurance.

### The oART workflow, data collection and analysis

In this study, *workflow* refers to the complete end-to-end oART procedure, while *process steps* denote its sequential components. The daily oART workflow on the Ethos system (versions 1.1 and 2.0) comprised eight process steps (Fig. 1).

**Fig. 1:**
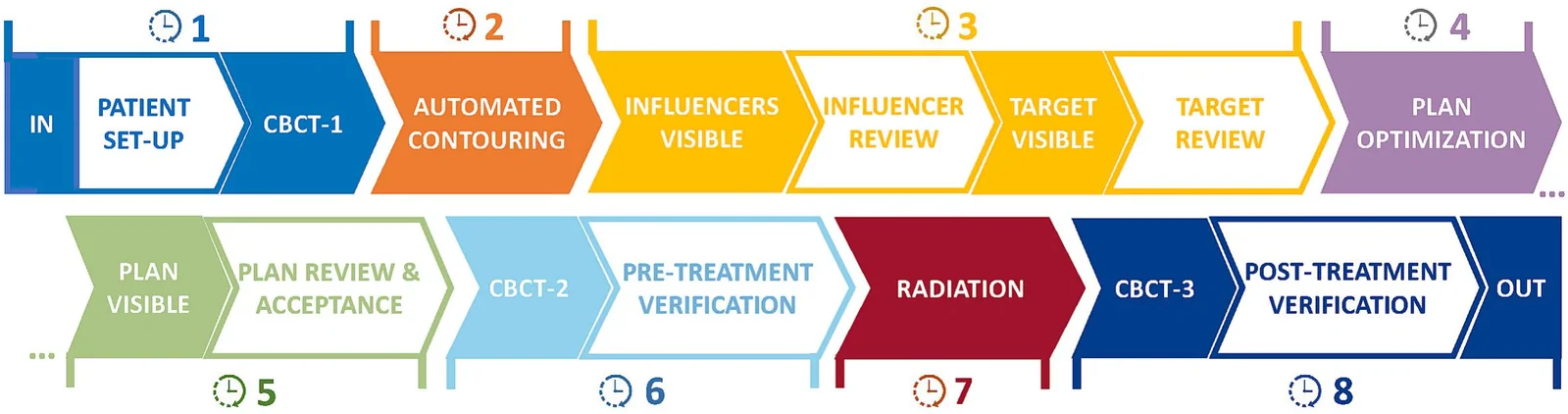
**. Schematic overview of the cervical oART workflow.** Eight sequential process steps, indicated by different colours, were used for process-level efficiency evaluation. The workflow started with patient entering the room, patient setup and CBCT acquisition (step 1), followed by AI-based contouring of influencer and target structures (step 2), sequential review and correction of influencer contours followed by target contours (step 3), generation of the scheduled plan by dose recalculation and the adapted plan by re-optimization (step 4), plan review, selection and approval including 3D dose verification (step 5), pre-treatment verification CBCT (CBCT-2) to assess patient positioning and target coverage, with corrective actions if required (step 6), treatment delivery (step 7), and post-treatment verification CBCT (CBCT-3) to verify that the uterus remained within the PTV and the patient left the treatment room (step 8).

Following CBCT acquisition, the organs were automatically contoured using AI. Contours of the so-called ‘influencers’ [16] uterus, rectum and bladder were manually corrected if deviations were more than a few mm, with particular emphasis on the uterus as it is the primary target. Target contours were propagated from the planning CT to the CBCT using deformable image registration. For CTV-T_LR_ contour-guided deformable registration was used using the influencers uterus, bladder, and rectum contours. In case where deviations extended outside the PTV, the CTV-E and CTV-T_LR_ contours were manually corrected. However, in practice, when making adjustments, CTV-T_LR_ contours that technically still fell within the PTV were often included in the correction as well. The user could then select the adapted plan, which was optimized using the same clinical goals and beam setup as the reference plan, or the scheduled plan, which was the reference plan recalculated on the daily planning CBCT. After plan review, a position pre-treatment verification CBCT (CBCT-2) was acquired before treatment delivery, to confirm patient positioning and to visually verify that the uterus was within the PTV. Following treatment delivery, a post-treatment verification CBCT (CBCT-3) was acquired to verify that the uterus had remained within the PTV, after which the patient left the treatment room. CBCT-3 was not acquired in sessions involving multiple isocentres, due to technical limitations.

Workflow feasibility was defined as the ability to complete the daily online adaptive workflow within routine clinical practice while maintaining target coverage. This was evaluated by assessing treatment session duration, workflow events, and dosimetric comparisons.

Treatment session duration and individual process step times were derived from DICOM metadata, supplemented with Ethos and manual timestamps where required. Workflow events were identified from treatment records and included contour editing, plan selection, patient positioning, workflow interruptions, bladder emptying, rectal venting, and extension of any part of the uterus outside the PTV before and after treatment (Fig. 3).

Per-session dosimetric comparisons between reference, scheduled, and adapted plans were performed using predefined DVH metrics for CTV-T_LR_ (D_98%_), bladder and rectum (V_30Gy_ / V_40Gy_), as these structures were consistently reviewed and corrected during each session.

### Statistics

Statistical analysis of total session duration and individual process step durations was performed at both the patient and session level. Within-patient comparisons were conducted by calculating, per patient, the median duration of the first five treatment sessions and comparing these with the median duration of sessions 6–25 using paired Wilcoxon signed-rank tests. All timing variables are reported as medians with interquartile ranges (IQRs), and two-sided *p*-values <0.05 were considered statistically significant. Spearman rank correlation analysis was applied at the session level to assess monotonic associations between individual process step durations and total session duration.

## Results

29 of 30 patients received the standard schedule of 25 fractions. One patient (patient 6) received 19 fractions, having initially started treatment with a LoP approach for logistical reasons before transitioning to oART on the Ethos system. In total, 744 oART sessions were evaluated.

### Timing evaluation

Fig. 2 provides an overview of the duration and variability of individual oART session process steps, as well as the total session duration across all patients. The median total session duration across all patients was 33.0 min (IQR 13.0 min). The patient setup and first CBCT (step 1) showed a median duration of 5.0 min (IQR 3.5 min) with considerable variability. Automated contouring (step 2) was completed in a median time of 1.4 min (IQR 0.5 min). Influencer and target review (step 3) demonstrated high variability, with a median duration of 10.0 min (IQR 8.3 min). Plan optimization (step 4), and evaluation and acceptance, including the secondary dose check (step 5), required median time of respectively 2.6 (IQR 1.4 min) and 3.7 min (IQR 1.7 min). The CBCT-2 verification (step 6) had a median duration of 1.8 min (IQR 1.1 min), but was prone to delays, particularly when patients needed to be taken off the treatment couch. Beam-on time (step 7) had a median duration of 2.7 min (IQR 1.4 min). Finally, CBCT-3 verification (step 8) was performed in 28 of 30 patients, with a median duration of 3.4 min (IQR 1.8 min).

**Fig. 2:**
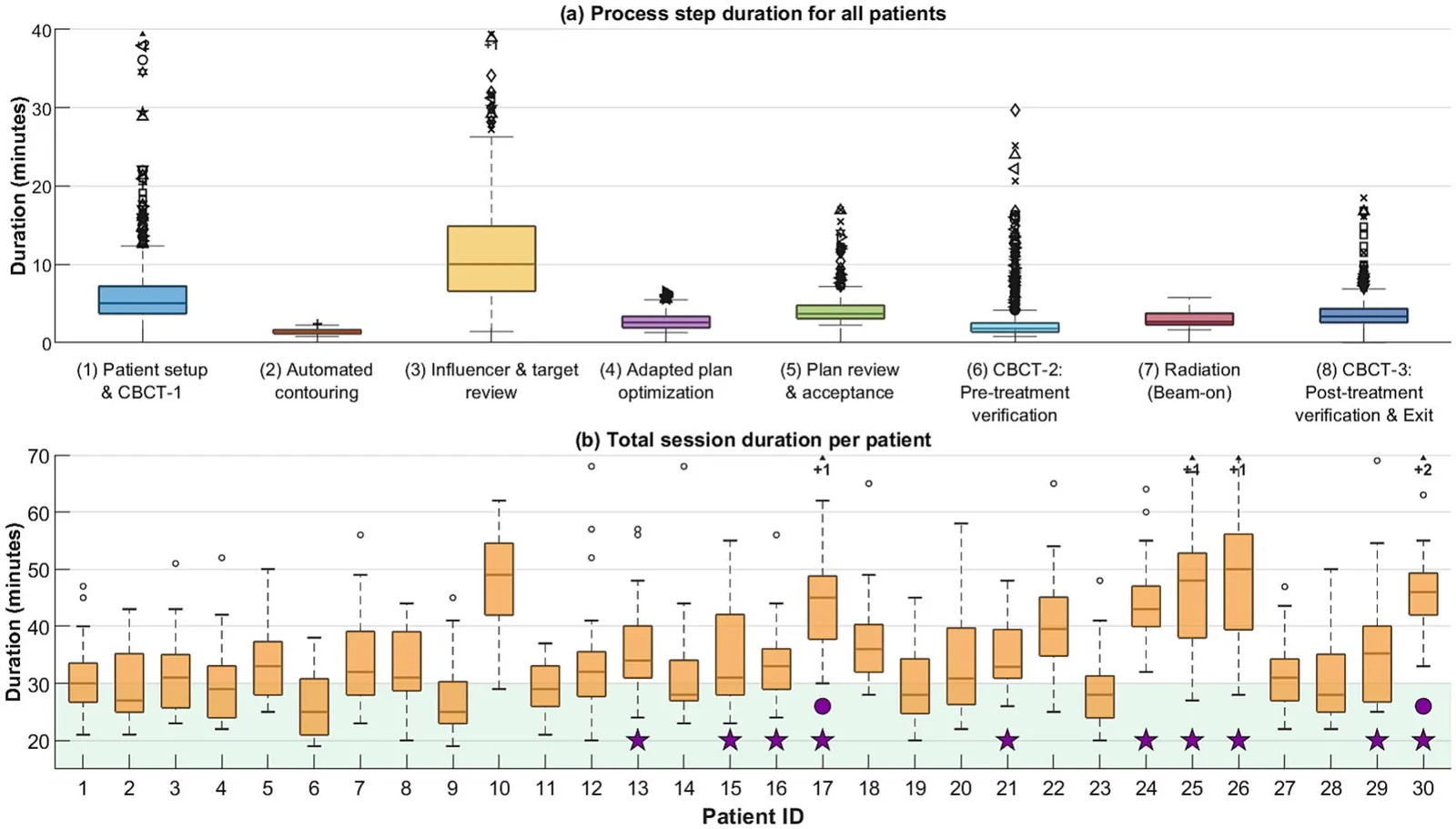
. **Cervical oART session duration.** (a) Process-step durations across all sessions, displayed as boxplots. Outliers are indicated with a different symbol for each individual patient. (b) Total session duration per patient. The green bar indicates a 30-min treatment slot. Session outliers are indicated with an open circle. Patients are numbered chronologically according to treatment order. Purple stars indicate patients with SIB lymph nodes and purple circles indicate patients with two isocentres. (For interpretation of the references to colour in this figure legend, the reader is referred to the web version of this article.)

The duration of individual process steps across all sessions demonstrated substantial inter- and intra-patient variability. Imaging and beam delivery steps showed relatively stable durations, whereas decision-intensive steps exhibited the greatest temporal variation.

Paired within-patient comparison showed no significant difference in median total session duration between the first five supervised sessions and the subsequent 20 RTT-led sessions (32.5 versus 32.0 min). Spearman rank correlation analysis at the session level demonstrated a strong positive association between the duration of influencer and target evaluation (step 3) and total session duration (ρ = 0.73, *p* < 0.001). A modest but statistically significant association was also observed between step 3 and CBCT-2 verification (step 6) duration (ρ = 0.27, *p* < 0.001). In contrast, other workflow steps showed weak correlations with total session duration.

### Workflow events evaluation

Predefined workflow events across all 744 sessions are summarized in Fig. 3, with patient-level distributions of selected events shown in Fig. 4. Influencer and target contours required manual correction in all patients. The scheduled plan was selected once because the adapted plan resulted in unacceptable extension of the 95% isodose outside the PTV.

**Fig. 3:**
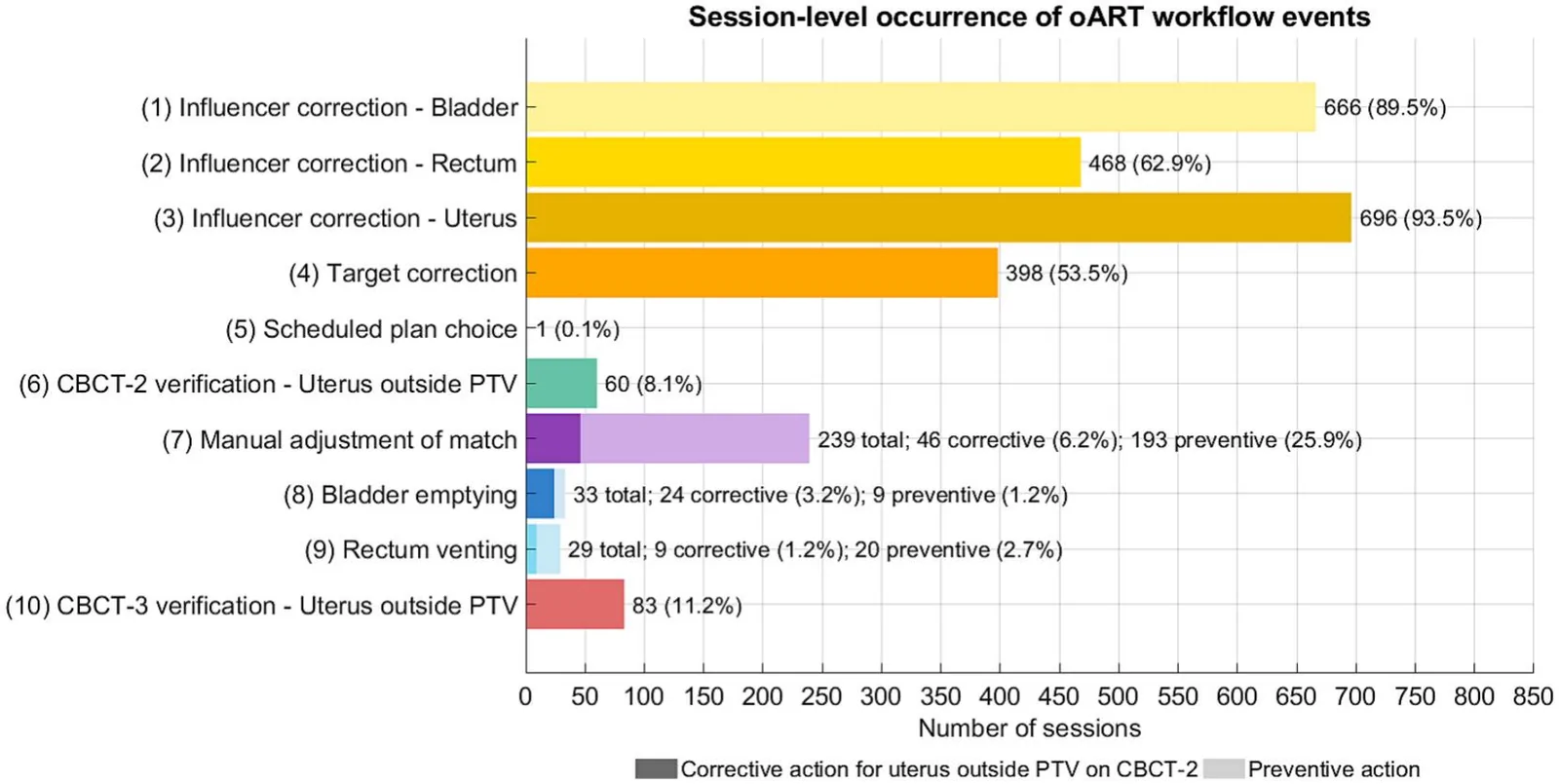
**Occurrence of predefined oART workflow events during 744 sessions.** Events included manual correction of AI-generated influencer contours for the bladder, rectum, and uterus [1], [2], [3]; manual correction of propagated target contours [4]; selection of the scheduled rather than the adapted plan [5]; extension of any part of the uterus outside the PTV on CBCT-2 [6]; manual adjustment of the image match [7]; temporarily taking the patient off the treatment couch for a workflow intervention for bladder emptying [8] and/or rectal venting [9]; and extension of any part of the uterus outside the PTV on CBCT-3 [10]. For events 7, 8, and 9, darker bar segments indicate corrective actions performed when part of the uterus extended beyond the PTV on CBCT-2, whereas lighter segments indicate preventive actions performed while the uterus was still within the PTV to reduce the risk of the target moving outside the PTV before or during treatment.

**Fig. 4:**
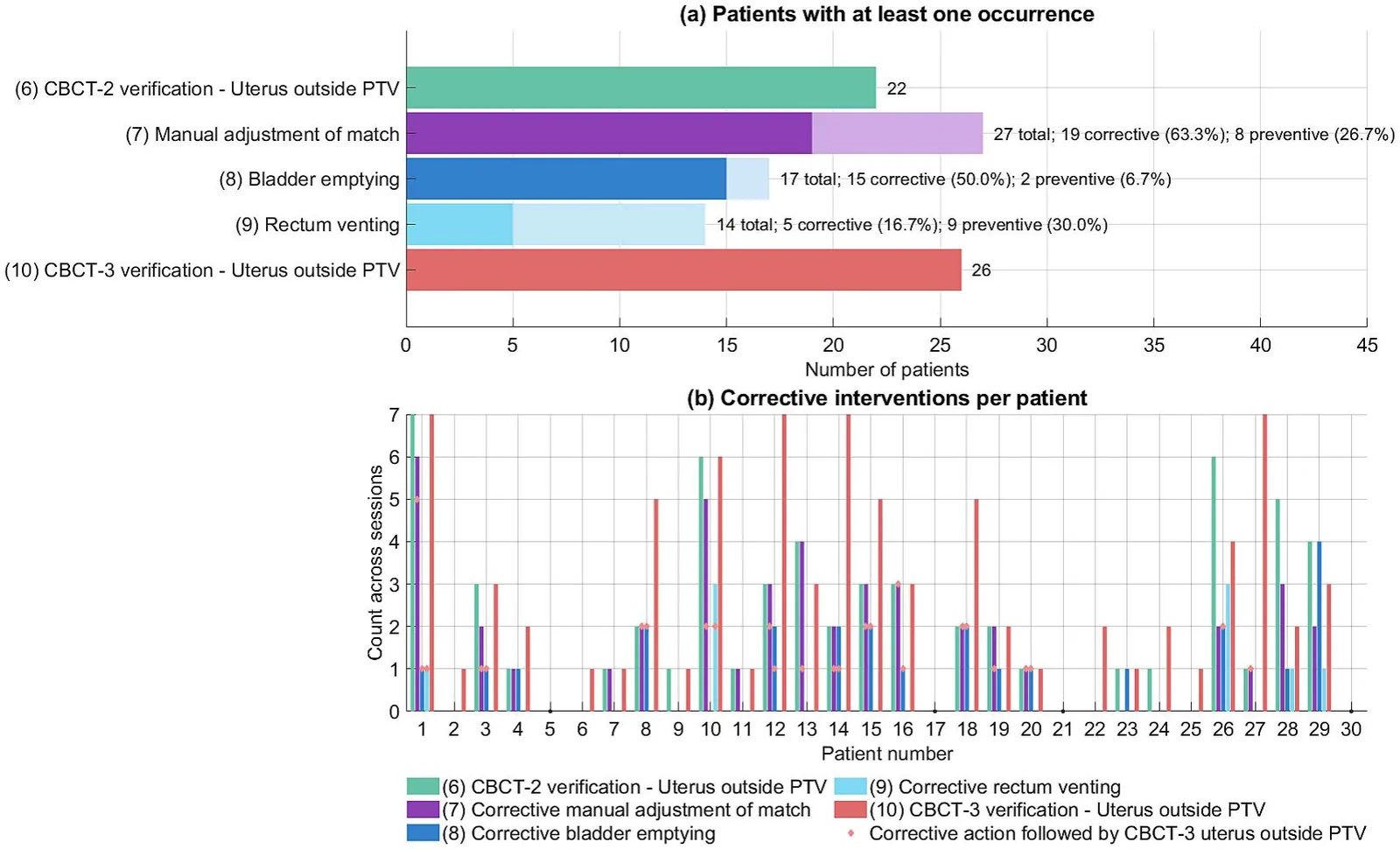
**. Patient-level occurrence of selected workflow events related to uterine position and workflow interventions.** (a) Number of patients with at least one occurrence of extension of part of the uterus outside the PTV on CBCT-2 [6], manual adjustment of the image match [7], bladder emptying [8], rectal venting [9], or extension of any part of the uterus outside the PTV on CBCT-3 [10]. (b) Number of occurrences of these events per patient. For events 7, 8, and 9, darker bar segments indicate corrective actions performed in sessions in which part of the uterus extended beyond the PTV on CBCT-2, whereas lighter segments indicate preventive actions performed while the uterus was still within the PTV to reduce the risk of the target moving outside the PTV before or during treatment. Red diamonds indicate the number of sessions in which a corrective action was performed, and a part of the uterus was also outside the PTV on CBCT-3. (For interpretation of the references to colour in this figure legend, the reader is referred to the web version of this article.)

Actions were classified as corrective when initiated because the uterus was outside the PTV on CBCT-2, and as preventive when performed to avoid subsequent displacement or because of excessive bladder filling or rectal gas. Corrective and preventive actions comprised 46 and 193 manual match adjustments, 24 and 9 bladder-emptying interventions, and 9 and 20 rectal-venting interventions, respectively. The uterus was outside the PTV on CBCT-2 in 60 sessions (8.1%) and on CBCT-3 in 83 sessions (11.2%). Following any corrective action, it remained outside the PTV on CBCT-3 in 27 of 57 sessions (Fig. 4b). If the patient was taken off the couch for bladder emptying, the patient was repositioned and the workflow resumed at the point where it had been paused. However, in five sessions the cervix-uterus was too far outside the PTV and the workflow was restarted.

Fig. 5 shows a representative patient illustrating workflow-related interventions between CBCT-1 and CBCT-2. In one session, increased bladder filling combined with changes in rectal gas between CBCT acquisitions resulted in extension of a part of the uterine fundus outside the PTV, leading to bladder emptying prior to irradiation. In another session of the same patient, CBCT-2 demonstrated extensive rectal and bowel gas occupying the PTV, while uterine coverage remained adequate. After consultation of an MPE according to the traffic-light protocol, rectal venting was performed. Repeat CBCT-2 confirmed clearance of rectal gas. Despite increased bladder filling, uterine position remained within the PTV, allowing treatment delivery.

**Fig. 5:**
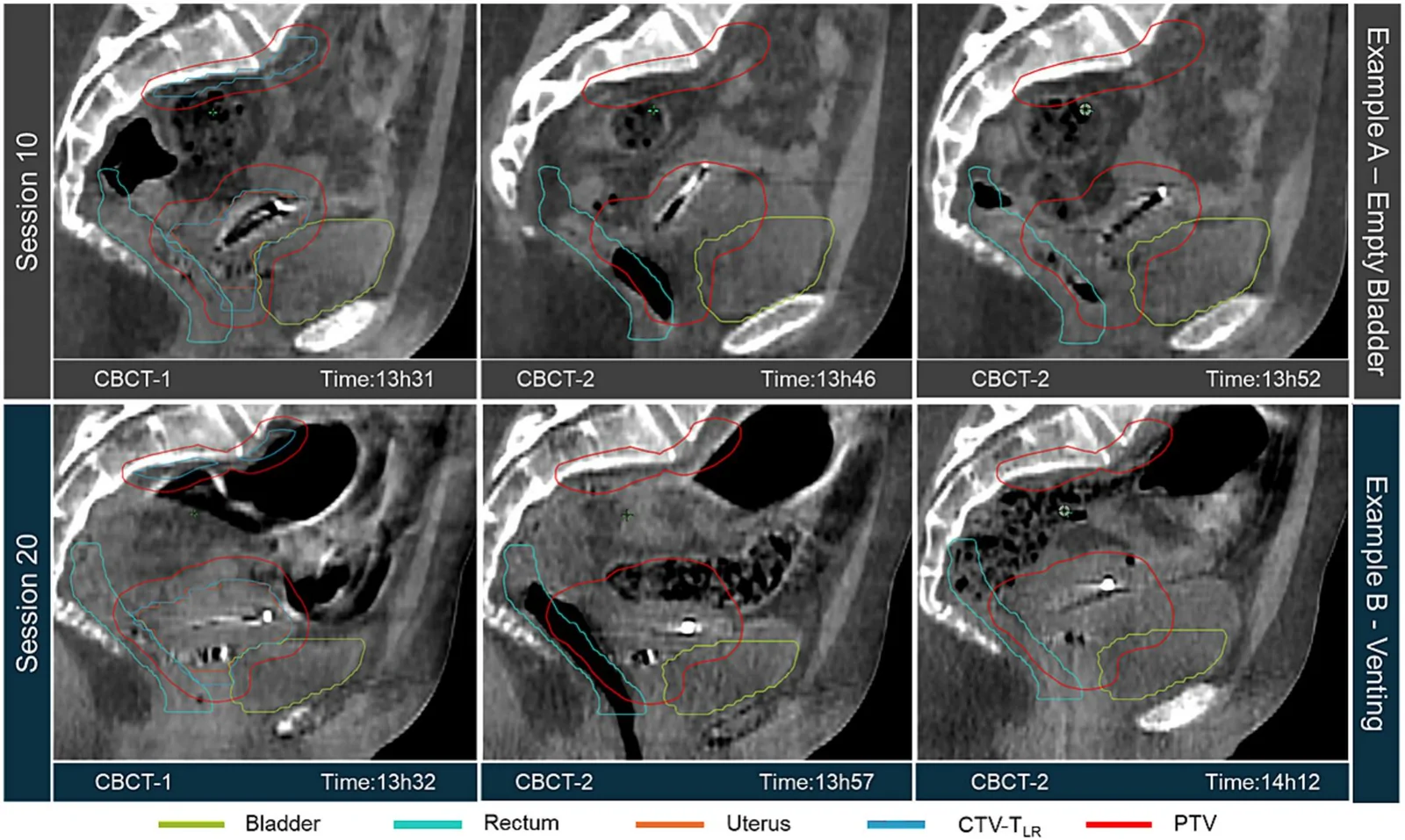
**Example from patient 12 illustrating two workflow-related events occurring between CBCT-1 (process step 1) and CBCT-2 (process step 6).** (a) During session 10, increased bladder filling and rectal gas resulted in extension of part of the uterus outside the PTV, involving the uterine fundus. As a corrective action, the patient was requested to empty her bladder prior to treatment. (b) During session 20, substantial rectal and bowel gas was present on the CBCT-2, with a large volume of air and bowel located within the PTV while the uterus itself remained within the PTV. Rectal venting was therefore performed prior to treatment. Bladder, rectum, uterus, CTV-T_LR_, and PTV contours shown are based on CBCT-1. The illustrated patient had an intrauterine device in place during EBRT, which had no clinical consequences and was removed prior to brachytherapy.

Target coverage of the adapted plans was maintained at ≥95% CTV-T_LR_ D_98%_ across all fractions, with minimal intra-patient variation. As expected, scheduled plans showed substantial variability, with several patients exhibiting underdosage of CTV-T_LR_ (supplementary fig. S2). V_30Gy_ and V_40Gy_ of bladder and rectum of the adapted plan also varied markedly between patients, largely reflecting differences in bladder volume, while rectum dose was more stable (supplementary fig. S3).

## Discussion

In this two-year, single-center study, we evaluated the implementation of CBCT-guided oART for cervical cancer by assessing workflow efficiency and the feasibility of an RTT-led workflow.

### Workflow efficiency

Our median total session duration of 33 min is comparable to previously reported CBCT-guided cervical oART workflows. Wang et al. [20] reported adaptive workflow of approximately 22–23 min, Shelley et al. [19] described 29 min from CBCT acquisition to beam-off, and Branco et al. [18] reported total session durations of around 30 min. Most published studies describe only part of the workflow, whereas our in-room time included the complete process from patient entrance to exit. Further improvement of the workflow efficiency would be required to reduce the total session time to 20 min, which was the duration of the time slot for LoP that was used in our clinic before the introduction of oART.

Session duration was primarily driven by the decision-intensive steps, particularly contour review and CBCT-2 verification, rather than imaging or treatment delivery. The absence of a learning curve within patients indicates that, following structured training, an RTT-led workflow can be performed with consistent efficiency.

### Workflow events

In our cohort, manual correction of AI-generated influencer and target contours was performed in most sessions. This is consistent with findings by Zhang et al. [30], who showed that although AI-generated contours are generally clinically acceptable, adapted plans based on fully unedited contours underperformed compared with plans optimized on manually corrected structures, particularly for highly mobile targets such as the uterus and vaginal cuff [30]. However, this does not imply that every individual contour edit is dosimetrically relevant. It remains unclear which specific edits are required to ensure adequate plan quality versus those that primarily add workflow burden.

Consistent with this uncertainty, influencer and target review (step 3) was the strongest contributor to total session duration and was significantly associated with prolonged CBCT-2 verification (step 6). Together, these findings highlight contour review as a key target for workflow optimization. Manual contour corrections should therefore be primarily reserved for situations where PTV coverage is compromised. Wang et al. [31] compared propagated lymph node boost CTVs with the corrected, clinically accepted contours and found that the 95% Hausdorff distance was larger than 30 mm in 3.7% of the nodal boost CTVs, which indicates that considerable contour corrections are still required. Despite daily adaptation, CBCT verification occasionally showed extension of part of the uterine fundus outside the PTV, distant from the GTV, most often related to intrafraction bladder filling or rectal distension, particularly during longer adaptation times. Pre-treatment findings were managed according to the traffic-light protocol, while post-treatment findings were reviewed with the radiation oncologist to determine whether any adaptations to subsequent treatment sessions were warranted. Similar intrafraction motion has been reported previously, particularly in patients with a small initial bladder volume [6].

In our cohort, an adapted plan was selected in all but one session, indicating that daily adaptation with reduced margins maintained robust CTV coverage and OAR sparing. ITV volumes were not generated: Instead, reduced CTV-to-PTV margins were applied compared with our LoP approach. The observed intrafraction uterine motion did not indicate a need to modify these margins, as the applied margins provided the intended geometric coverage [32], [33]. Furthermore, EMBRACE II permits limited cumulative underdosage of the non-involved uterus, acknowledging the contribution of subsequent brachytherapy to the total uterine dose. Similarly, Wang et al. reported maintained target coverage and low toxicity using comparable uterine and nodal margins despite residual intrafraction variability [21].

### Team training and workflow organization

Successful implementation was supported by structured training, clear multidisciplinary protocols, and on-call expert support, building on our previously reported RTT-led bladder oART implementation [17]. Transition to an RTT-led workflow was feasible following competency-based training. Regular feedback, clear contour and escalation guidelines, offline checks and effective team communication remain essential to maintain workflow quality and patient safety [34], [35].

### Strengths, limitations, and future directions

The main strength of this study is the evaluation of a real-world clinical implementation using systematic workflow analysis. Inclusion of a heterogeneous patient population increases clinical relevance, although more complex treatments, such as two-isocentre cases, were relatively underrepresented. The phased inclusion of patients with increasing complexity of the target volume, the limited cohort size, and minor uncertainty in manually recorded timestamps reduced statistical power and limited assessment of learning effects. Concurrent system upgrades and AI improvements, and expansion of clinical indications prevented evaluation of their individual impact. Intrafraction motion was analysed only qualitatively, by counting reported workflow events about extension of the uterus outside the PTV, limiting the evaluation of the PTV margin that was used in this study.

Future workflow optimization should focus on improving AI contouring accuracy and speed, reducing manual edits, accelerating plan calculation, and automated post-treatment CBCT analysis to better characterize intrafraction motion. Given the growing evidence supporting reduced margins with daily oART, future prospective studies should determine whether patient-specific PTV margin reduction translates into reduced toxicity, improved quality of life while maintaining target coverage.

## Conclusion

Daily CBCT-guided cervical oART can be efficiently implemented within an RTT-led workflow while maintaining target coverage despite intra-fraction anatomical variability. Workflow efficiency and workflow event analysis demonstrated that routine clinical implementation is feasible.

The following are the supplementary data related to this article.

**Supplementary Fig. S1:**
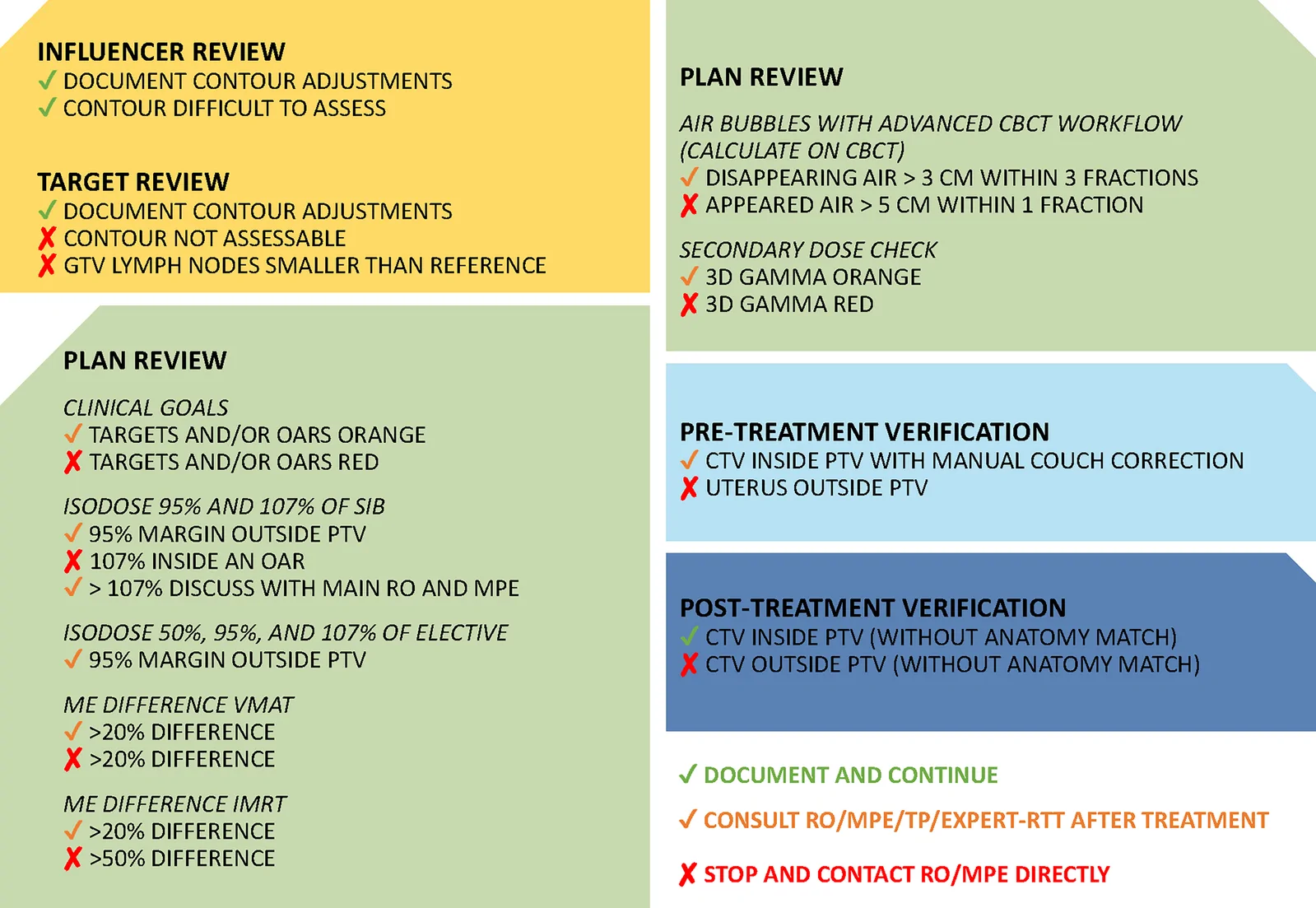
**Traffic-light protocol for RTT-Led cervical oART workflow.** The traffic-light protocol covers influencer review, target review, plan review (clinical goals, isodose coverage, monitor unit differences, air cavity evaluation, secondary dose verification), and pre- and post-treatment verification. Colour-coded decision boxes indicate required actions: green checkmarks denote acceptance and continuation, orange checkmarks indicate consultation with the radiation oncologist (RO), medical physics expert (MPE), technical physician (TP) or oART expert radiation therapist (expert-RTT) after treatment, and red crosses indicate criteria requiring immediate interruption and direct consultation of RO or MPE. Thresholds for geometric, dosimetry, and anatomical deviations are explicitly defined to standardize decision-making and ensure treatment safety and consistency. (For interpretation of the references to colour in this figure legend, the reader is referred to the web version of this article).

**Supplementary Fig. S2:**
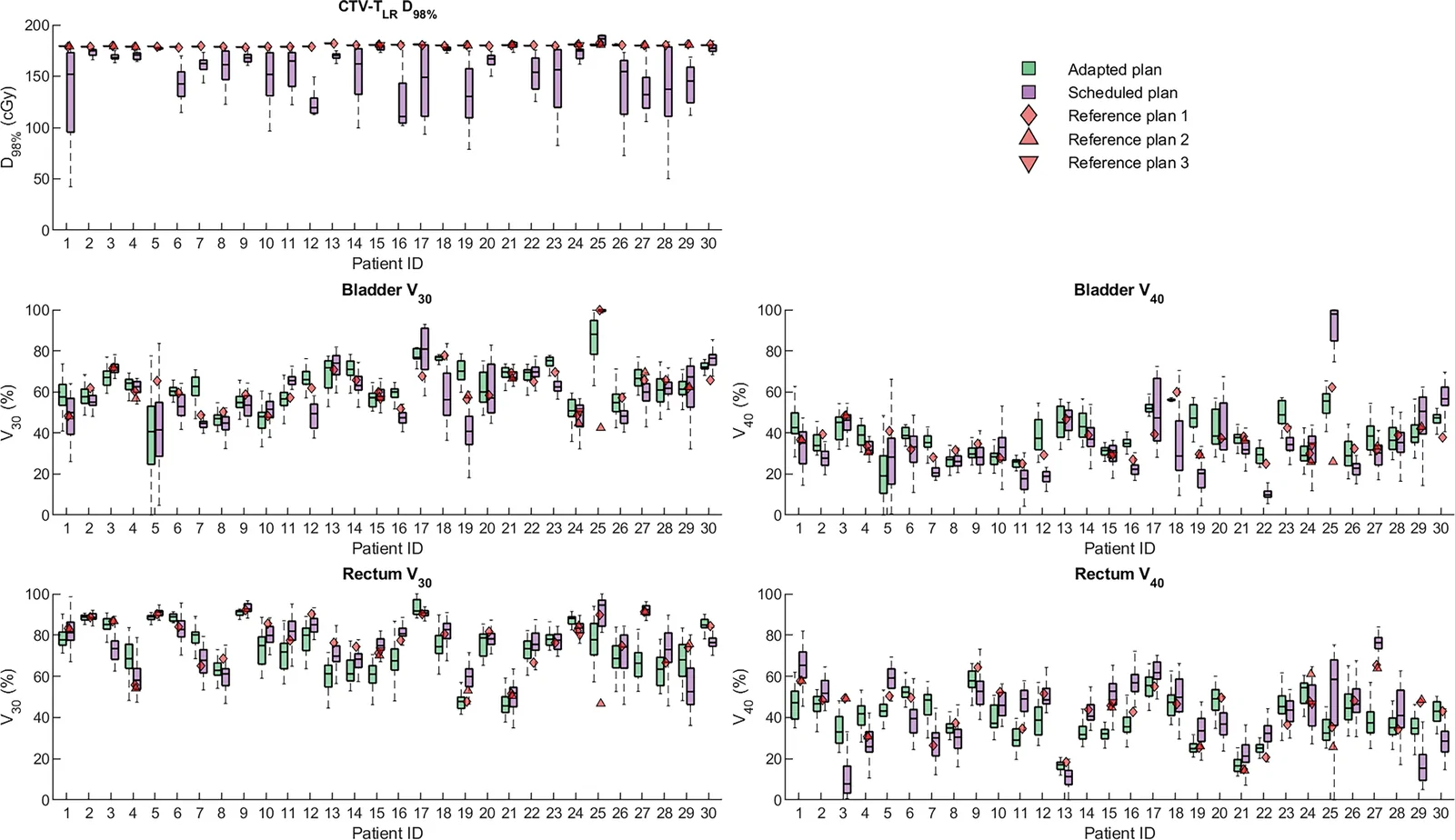
**Dose–volume histogram parameters for CTV-T**_**LR**_**(D**_**98%**_**), bladder and rectum (V**_**30**_**and V**_**40**_**) across all fractions and patients.** Boxplots represent scheduled (purple) and adapted (green) plans per patient in dose per fraction for all sessions. Red diamonds and triangles show the reference plan(s). (For interpretation of the references to colour in this figure legend, the reader is referred to the web version of this article).

**Supplementary Fig. S3:**
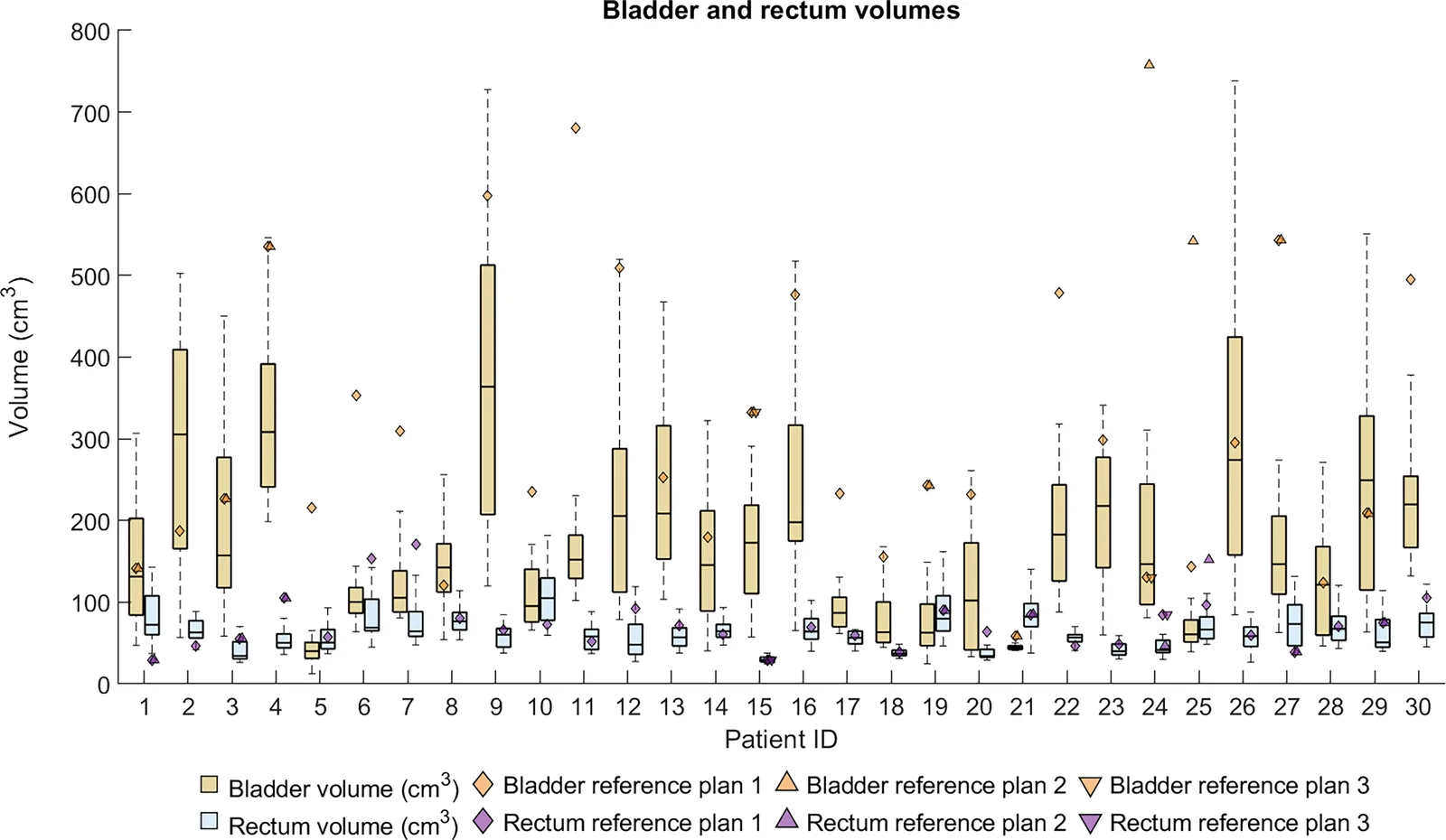
**Bladder and rectum volumes across all fractions and patients**. Boxplots per patient (x-axis) show inter- and intra-patient variability in bladder (yellow) and rectum (light blue) volumes (cc). Red diamonds and triangles show the bladder and rectum volume in the reference plan(s). (For interpretation of the references to colour in this figure legend, the reader is referred to the web version of this article).

Supplementary material 4

## Declaration of generative AI and AI-assisted technologies in the manuscript preparation process

During the preparation of this work, the authors used ChatGPT (OpenAI) and the Amsterdam UMC AI Proeftuin (a secure institutional generative AI environment) to assist with language editing, improving readability, and reducing manuscript length. After using these tools, the authors critically reviewed and edited the content as needed and take full responsibility for the content of the published article.

## CRediT authorship contribution statement

**Danique L.J. Barten:** Writing – review & editing, Writing – original draft, Visualization, Validation, Project administration, Methodology, Investigation, Formal analysis, Data curation, Conceptualization. **Karin Goudschaal:** Writing – review & editing, Methodology, Data curation, Conceptualization. **Karel A. Hinnen:** Writing – review & editing, Data curation, Conceptualization. **Rianne de Jong:** Writing – review & editing. **Jan Wiersma:** Writing – review & editing, Resources, Data curation. **Niek van Wieringen:** Writing – review & editing. **Zdenko van Kesteren:** Writing – review & editing. **Arjan Bel:** Writing – review & editing, Supervision. **Angelique van Vlaenderen:** Writing – review & editing. **Sandy Loopeker:** Writing – review & editing. **Bradley R. Pieters:** Writing – review & editing, Data curation, Conceptualization. **Jorrit Visser:** Writing – review & editing, Validation, Supervision, Methodology, Data curation, Conceptualization. **Colette B.M. van den Broek:** Writing – review & editing, Validation, Supervision, Methodology, Data curation, Conceptualization.

## Ethics declaration

Informed consent and patient details**:** Written informed consent to take part in the study and to publish the article has been obtained from all participants or their legal representatives. The privacy rights of participants have been observed.

Studies in Human: This study was performed in compliance with relevant laws, regulatory frameworks and guidelines where the research took place. This study was approved by the Medical Ethics Committee of Amsterdam UMC. (Approval No. 2025.0921)

## Funding

None

## Declaration of competing interest

The authors declare that they have no known competing financial interests or personal relationships that could have appeared to influence the work reported in this paper.
