# Supplementary material for: Cone beam computed tomography guided online adaptive radiotherapy for cervical Cancer: Two-year single-Centre implementation and clinical experience
Source: Tech Innov Patient Support Radiat Oncol. 2026 Sep 27;40:100442. doi: 10.1016/j.tipsro.2026.100442 (PMC13658862; doi:10.1016/j.tipsro.2026.100442)
Supplement: Supplementary file 1 [file mmc4.docx]

**Table A1	Summary of patients characteristics: Patient ID, age while treated, Tumor-Node-Metastasis (TNM) stage (clinical), Body Mass Index (BMI), Histopathology, and clinical target volume low-risk (CTV-T_LR_) (cm³)**

| Patient ID | Age | TNM | BMI | Histopathology | Volume  CTV-T_LR_ (cm3) |
| --- | --- | --- | --- | --- | --- |
| P01 | 31 | FIGO Stage IIB, cT2bN0M0 | 22 | Squamous cell carcinoma | 116.8 |
| P02 | 67 | FIGO Stage IIB and IIA2, cT2bN0M0 | 25 | Adenocarcinoma | 209.0 |
| P03 | 35 | FIGO Stage IB2, cT1b2N0M0 | 37 | Squamous cell carcinoma | 201.6 |
| P04 | 75 | FIGO Stage IB1, cT1b1N0M0 | 31 | Adenocarcinoma | 184.6 |
| P05 | 39 | FIGO Stage IIIB, cT3bN0M0 | 22 | Squamous cell carcinoma | 228.9 |
| P06 | 67 | FIGO Stage IIA2, cT2a2N0M0 | 27 | Squamous cell carcinoma | 260.9 |
| P07 | 81 | FIGO Stage IIA1, cT2a1N0M0 | 28 | Squamous cell carcinoma | 192.7 |
| P08 | 72 | FIGO Stage IIIB, cT3bN0M0 | 22 | Adenocarcinoma | 103.3 |
| P09 | 31 | FIGO Stage IB2, cT1b2N0M0 | 20 | Squamous cell carcinoma | 239.6 |
| P10 | 82 | FIGO Stage IB2, cT1b2N0M0 | 26 | Squamous cell carcinoma | 145.8 |
| P11 | 39 | FIGO Stage IB2, cT1b2N0M0 | 36 | Adenocarcinoma | 157.5 |
| P12 | 28 | FIGO Stage IB2, cT1b2N0M0 | 21 | Adenocarcinoma | 100.9 |
| P13 | 60 | FIGO Stage IIA1, cT2a1N0M0 | 30 | Squamous cell carcinoma | 108.2 |
| P14 | 59 | FIGO Stage IB2, cT1b2N0M0 | 28 | Squamous cell carcinoma | 87.9 |
| P15 | 47 | FIGO Stage IIB, cT2bN0M0 | 24 | Squamous cell carcinoma | 174.6 |
| P16 | 36 | FIGO Stage IIB/IIIB, cT3bN0M0 | 23 | Squamous cell carcinoma | 223 |
| P17 | 79 | FIGO Stage IVA, cT2bN1M1a | 23 | Undifferentiated carcinoma | 226.8 |
| P18 | 58 | FIGO Stage IIB, cT2bN0M0 | 29 | Squamous cell carcinoma | 652.6 |
| P19 | 73 | FIGO Stage IIB, cT2bN0M0 | 28 | Squamous cell carcinoma | 74.6 |
| P20 | 72 | FIGO stage IIA1/IIB, cT2bN0M0 | 19 | Adenocarcinoma | 104.9 |
| P21 | 58 | FIGO stage IIIC1, cT3bN1M0 | 27 | Squamous cell carcinoma | 94.4 |
| P22 | 87 | FIGO stage IIIA, cT3aN0M0 | 42 | Adenocarcinoma | 179.1 |
| P23 | 40 | FIGO stage IIA2, cT2a2N0M0 | 26 | Adenocarcinoma | 199.5 |
| P24 | 40 | FIGO stage IIIC1, cT3bN1M0 | 20 | Squamous cell carcinoma | 302.7 |
| P25 | 36 | FIGO stage IIIC1, cT2bN1M0 | 26 | Squamous cell carcinoma | 409.9 |
| P26 | 61 | FIGO stage IIIC1, cT2bN1M0 | 18 | Squamous cell carcinoma | 102.9 |
| P27 | 58 | FIGO stage IIB/IIA, cT2bN0M0 | 22 | Adenocarcinoma | 145.9 |
| P28 | 59 | FIGO stage IIIB, cT3bN0M0 | 23 | Adenocarcinoma | 237.6 |
| P29 | 33 | FIGO stage IIIC1, cT1b2N1M0 | 29 | Squamous cell carcinoma | 204.2 |
| P30 | 43 | FIGO stage IIIC2, cT3bN1M0 | 21 | Squamous cell carcinoma | 359.6 |
